# Supplementary material for: Verbal Encoding Deficits Impact Recognition Memory in Atypical “Non-Amnestic” Alzheimer’s Disease
Source: Brain Sci. 2022 Jun 28;12(7):843. doi: 10.3390/brainsci12070843 (PMC9313460; doi:10.3390/brainsci12070843)
Supplement: Supplementary file 1 [file brainsci-12-00843-s001.zip › brainsci-1778212-supplementary.pdf]

Supplementary Materials

CVLT-II-SF	Whole group (N=37)	Amnestic (N=14)	PCA (N=14)	lvPPA (N= 9)
Trial 1	-2.1 (1.4)	-2.2 (1.3)*	-1.4 (1.4)^	-2.8 (1.3)*^
Total Learning (Sum Trials 1-4)	-1.9 (1.5)	-2.6 (1.3)*	-1.2 (1.4)†	-2.1 (1.5)*
SDFR	-1.7 (1.4)	-2.1 (1.2)*	-1.3 (1.6)	-1.6 (1.0)*
LDFR	-1.4 (1.2)	-1.9 (1.1)*	-1.3 (1.4)	-1.0 (0.8)
LDCR	-1.8 (1.4)	-2.4 (1.3)*	-1.5 (1.6)*	-1.5 (1.4)*
d'	-1.05 (1.2)	-1.4 (1.2)	-1.0 (1.3)	-0.7 (0.8)
C	-0.7 (1.4)	-1.3 (1.5)	0.04 (1.0)†	-0.7 (1.4)

SM Table S1. Demographically-adjusted CVLT-II-SF performance. Mean (SD) of demographically-adjusted z-scores presented for each measure. SDFR = Short Delay Free Recall. LDFR = Long Delay Free Recall. LDCR= Long Delay Cued Recall. d'= Recognition Discriminability d prime. C= Response Bias. * indicates impairment at the level of 1.5 SD below the mean or lower. † indicates differences between this group and the Amnestic Group at $p<0.05$. ^ indicates differences between PCA and lvPPA groups at $p<0.05$.

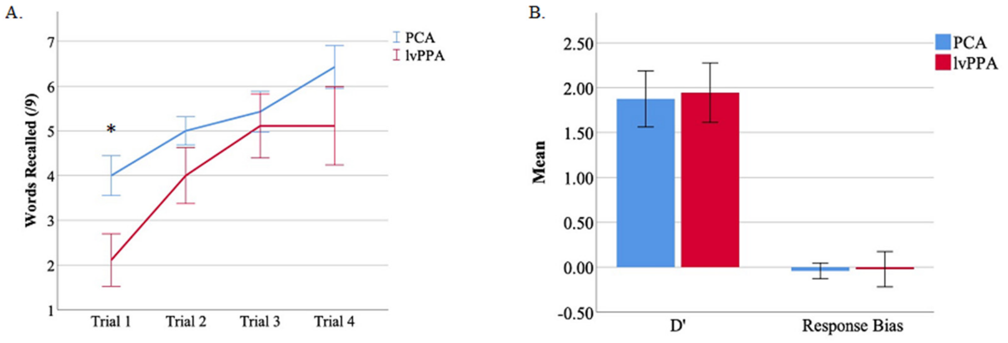

SM Figure S1. PCA and lvPPA are comparable on Total Encoding and Recognition Memory performance. **1A**. Non-Amnestic subtypes of AD—PCA and lvPPA—performed comparably on Total Learning (Sum of Trials 1-4; t=1.6, p=0.13, Cohen's d= 0.65) though lvPPA recalled fewer words at Trial 1 compared to PCA (t=2.60, p=0.02, Cohen's d= 1.10). **1B.** PCA and lvPPA groups were comparable (p>0.5) on Recognition Discriminability (D') and Response Bias.

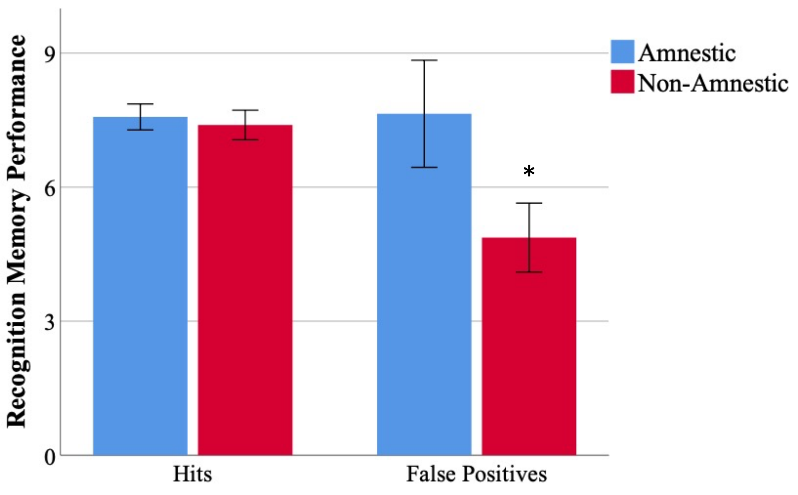

SM Figure S2. Recognition Memory: Hits versus false positives in Amnestic and Non-Amnestic AD. Given that d' is a measure encompassing both correctly identified target words (hits) as well as incorrectly endorsed foil words (false positives), we further investigated group differences on these specific metrics. We found that the groups performed similarly on hits (t=0.37, p=0.7) but the Non-Amnestic group endorsed fewer false positive items compared to the Amnestic group (t=0.20, p=0.05).

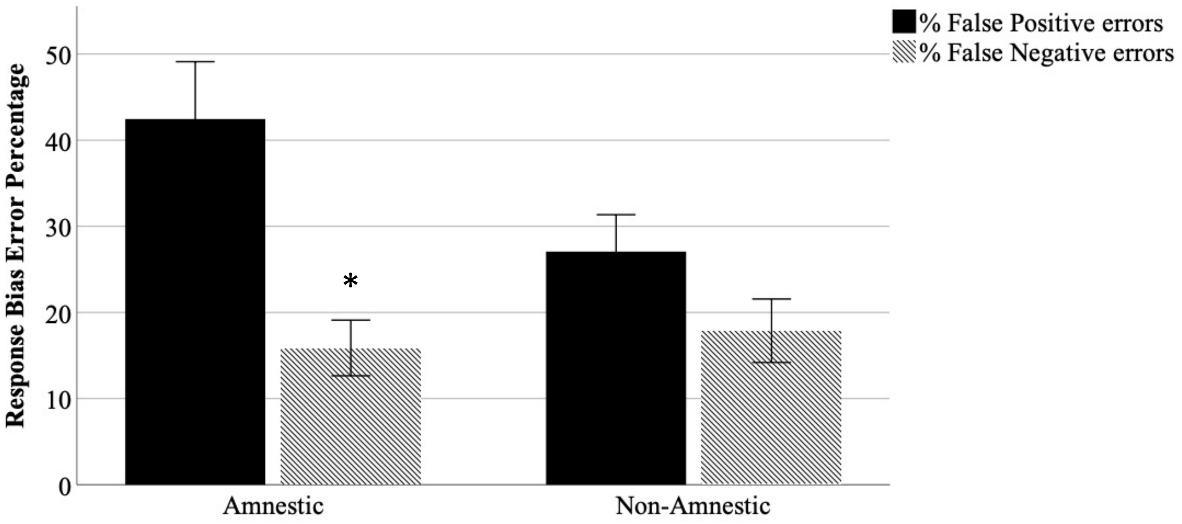

SM Figure S3. Recognition Memory: False positive versus false negative errors in Amnestic and Non-Amnestic AD. Analysis of the percentage of error type in recognition memory performance in the Amnestic group demonstrated a significantly higher percentage of false positive compared to false negative responses (t=3.3, p=0.006). In contrast, the Non-Amnestic group revealed only a trend level difference in the percentage of false positive errors compared to false negative errors (t=1.8, p=0.08; SM Figure 3).

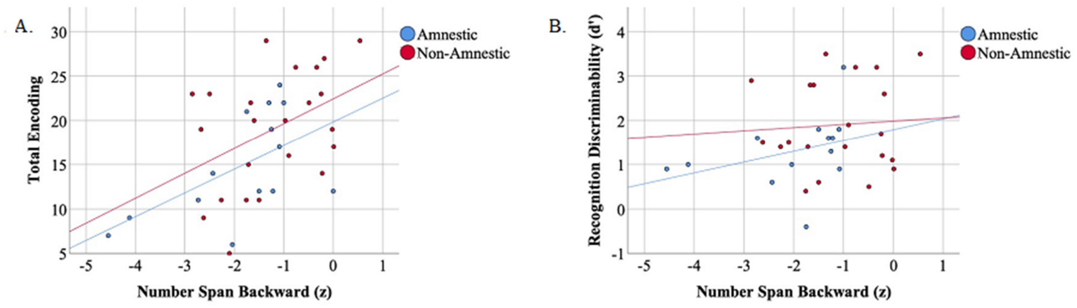

SM Figure S4. Working Memory is related to Total Encoding but not Recognition Discriminability. A. Working Memory, measured by Digit Span Backward, is related to Total Encoding in both Amnestic EOAD (*r*=0.56, *p*=0.04) and Non-Amnestic (*r*=0.42, *p*=0.04) groups. B. Digit Span Backward was not related to Recognition Discriminability (d') in either AD group.

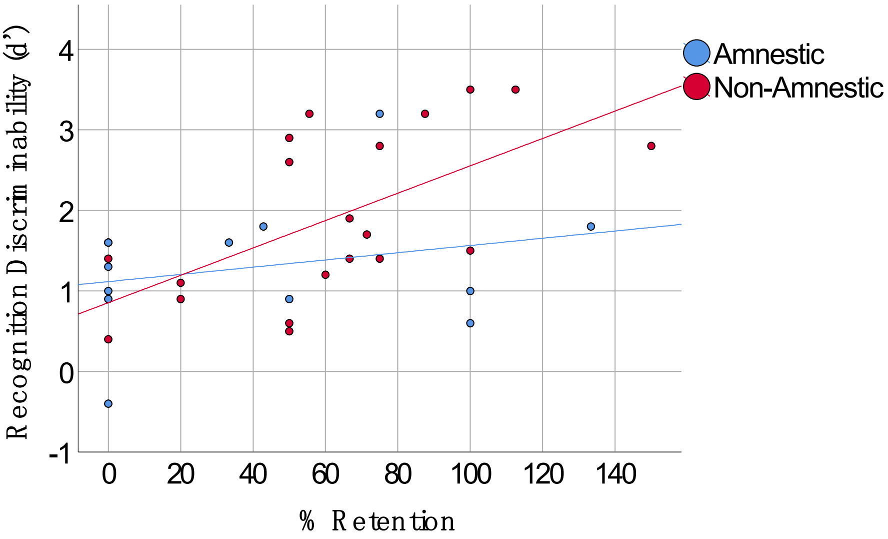

SM Figure S5. Percent retention is related to Recognition Discriminability (d') in Non-Amnestic AD. Total percent retention (long delay free recall / Trial 4 recall) is related to Recognition Discriminability (d') in the Non-Amnestic group (r=0.61, p=0.005), but not in the Amnestic group (p=0.49).

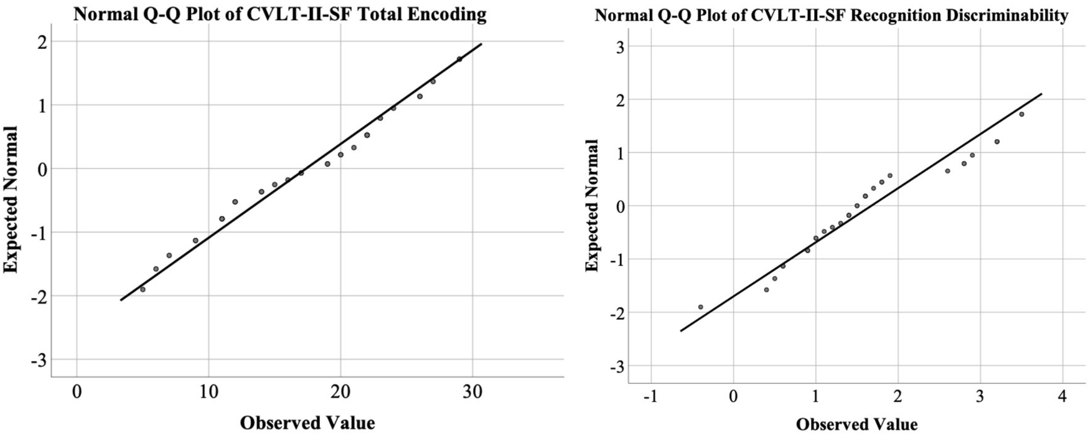

SM Figure S6. Normalization of variables of interest as shown with Q-Q plots. The data were largely normally distributed for the two main variables of interest on the CVLT-II-SF (Total Encoding and d’) based on the Shapiro-Wilk test being non-significant ($p<0.05$).
